# A Systematic Review on Childhood Non-Hodgkin Lymphoma: An Overlooked Phenomenon in the Health and Research Sector of Bangladesh

**DOI:** 10.7759/cureus.45937

**Published:** 2023-09-25

**Authors:** Tanzir Islam Britto, Shaikh Abdul Fattah, Mohammad Arif Ur Rahman, Mohammad Ashraf Uddin Chowdhury

**Affiliations:** 1 Nephrology, Chittagong Medical College Hospital, Chittagong, BGD; 2 Nephrology, National Institute of Kidney Diseases and Urology, Dhaka, BGD; 3 Medicine, Green Life Medical College, Dhaka, BGD; 4 Hematology, Dhaka Medical College Hospital, Dhaka, BGD; 5 Medicine, Centre for Medical Education, Directorate General of Health Services, Dhaka, BGD

**Keywords:** systematic review, bangladesh, pediatric oncology, non-hodgkin lymphoma (nhl), lymphoma

## Abstract

Globally, childhood cancer, particularly non-Hodgkin lymphomas (NHLs), is a prevalent concern. However, the difficulty becomes even more distressing in lower-middle-income nations such as Bangladesh. The insufficiency of research, resources, inadequate guidelines, expensive treatment costs, and specialized knowledge exacerbate the challenges associated with the treatment of certain types of cancers. Our investigation looked extensively into the circumstances prevailing in Bangladesh, with the objective of providing a comprehensive overview of the current status and approaches to managing NHL in the country. Through this work, our intention was to illuminate the domains that require immediate focus and assistance. To get insight into the present state of NHL in Bangladesh, our analysis focused on a selection of seven research articles and two case reports published between 2018 and 2023. In order to ensure the integrity and consistency of our review, we conducted a detailed selection procedure, employing the systematic PRISMA review methodology. From a pool of 294 papers, we selected the ones that met our predetermined criteria. These papers were sourced from reputable academic databases, such as Google Scholar and PubMed. The findings of our study indicate a higher prevalence of NHL among children in Bangladesh compared to Hodgkin lymphoma (HL). Additionally, this phenomenon exhibits a higher prevalence among male individuals. Our study revealed that in Bangladesh, there is a lack of a dedicated guideline or research center specifically focused on NHL. Additionally, the number of research centers and research dedicated to cancer treatment as a whole is limited. Our research aims to offer a complete analysis of NHL in the context of Bangladesh, with the intention of offering valuable guidance to healthcare professionals and policymakers. The utilization of our research outcomes has the potential to enhance patient care, facilitate the development of more effective clinical protocols, and promote greater accessibility and affordability of therapies. This has the potential to provide improved cancer care not only in Bangladesh but also in other comparable contexts worldwide.

## Introduction and background

Childhood cancer is becoming more common worldwide, and 84% of cancer cases in children under 15 years of age occur in low- and middle-income countries (LMIC) in Latin America, Africa, Asia, the Caribbean, and Polynesia [1,2]. Bangladesh, a small but overpopulated South Asian country, is also facing an alarming increase in the incidence of childhood cancer [3,4]. According to the World Child Cancer Report 2005, Bangladesh has 1.3 to 1.5 million childhood cancer patients [3]. In recent times, there has been a notable prevalence of pediatric cancer cases in Bangladesh, constituting a substantial proportion of the overall incidence [4]. Lymphoma is children's third most common malignant tumor [2,3]. It has a prevalence of about 7% of childhood cancers. Hodgkin’s (HL) and non-Hodgkin’s (NHL) are two types of lymphomas [1-3]. The majority of NHL in Bangladesh has emerged as a significant issue, as there has been a notable rise in reported cases recently [4].

Because of the absence or irregularity of the cancer register in Bangladesh, the actual cancer scenario, especially childhood cancer, is mostly unknown or misleading. Without authentic childhood cancer statistics or literature, the recent scenario is mostly hypothetical and irrelevant compared to Western countries [5,6]. By searching through the intricacies of NHL in the younger population, this review aims to equip readers with the necessary knowledge and insights to address the condition effectively. This review paper aims to summarize the available recent research papers obtained from two digital libraries, “Google Scholar” and “PubMed,” based on the NHL situation in Bangladesh. This review paper discussed the data collection procedures, treatments, etc., collected from the selected papers. Also, this paper collected information about the disease process, management, diagnosis, and expenses of NHL treatment in Bangladesh from other published articles, various official websites, and newspapers to depict the current situation in Bangladesh.

The remainder of this paper is organized as follows: Background section briefly describes the NHL, describing its clinical characteristics worldwide. In the Review section, we explained Preferred Reporting Items for Systematic Review and Meta-Analysis (PRISMA), which we have followed, wherein meticulous attention was given to the presentation of data related to gender distribution, the percentage of childhood NHL and HL, as well as the identification of the research centers participating. Furthermore, we presented an overview epidemiological analysis of NHL among children in Bangladesh, incorporating findings from nine scholarly articles. Additionally, we explored the healthcare administration, infrastructure, financial implications, and expenses related to the diagnosis and investigation of NHL inside the nation. Finally, the section Conclusion concludes the paper by highlighting the main findings of the papers, their limitations, and the scope for future work.

Background

Non-Hodgkin lymphoma (NHL), a collective term, denotes a list of malignant diseases originating from the body's immune cells, predominantly appearing as lymphadenopathy or solid tumors. NHL comprises a heterogeneous group of lymphoid neoplasms. About 70% of NHLs are higher-grade tumors with aggressive clinical behavior. Most of them are typically extra-nodal, with early widespread dissemination to the bone marrow and central nervous system (CNS) [3]. In children under the age of 15, NHL is the fifth most common type of pediatric cancer. Pediatric NHL, a malignancy that primarily affects individuals in their childhood and early adulthood, has emerged as a significant issue in the United States, with around 800 new cases reported annually and an incidence rate varying between 10 and 20 occurrences per one million individuals [7]. In developed nations, it accounts for about 7% of pediatric cancer cases [8]. This upward trend is largely attributed to the surge in adolescent NHL cases. Notably, the median age at diagnosis is around ten years, and the likelihood of developing pediatric NHL rises as children grow older [8-11].

The incidence and distribution of specific subtypes of NHL exhibit intriguing disparities across diverse populations, encompassing factors like age, race, and geographical location. Remarkably, the most prevalent subtypes of pediatric NHL trace their origins back to B-cell progenitors. The prevailing subtypes encompass Burkitt lymphoma (BL), diffuse large B-cell lymphoma (DLBCL), lymphoblastic T cell or B-cell lymphoma, and anaplastic large cell lymphoma [11]. Other subtypes, such as follicular lymphoma and marginal zone lymphoma, are less prevalent, making up approximately 7% of pediatric NHL cases. Both congenital and acquired immunodeficiency syndromes are linked to a heightened risk of NHL. Congenital immune deficiencies associated with NHL encompass common variable immunodeficiency, Wiskott-Aldrich syndrome, ataxia telangiectasia, and X-linked lymphoproliferative syndrome. These congenital immunodeficiencies can significantly influence treatment decisions [11].

During the assessment and diagnostic approaches of suspected children with NHL, some pathological conditions like vena cava obstruction, acute airway obstruction, intestinal obstruction, spinal cord compression, pericardial tamponade, lymphomatous meningitis, hyperuricemia, tumor lysis syndrome, ureteral obstruction, unilateral or bilateral hydronephrosis, venous thromboembolic disease, and a potential emergency complication of NHL may be present [12]. Because some of those are fetal and may cause death during the initial stage of treatment, those initial parameters are important and documented. The improved understanding of biology and the development of diagnostic tools have significantly led to the establishment of the World Health Organization's (WHO) Classification of Tumors of the Hematopoietic and Lymphoid Tissues [3]. The classification is as follows: lymphoblastic lymphoma (LBL/LL), Burkitt lymphoma (BL), anaplastic large-cell lymphoma (ALCL), and diffuse large B-cell lymphoma (DLBCL) [3].

Etiology

Non-Hodgkin lymphomas (NHL) are caused by infections, environmental stressors, immunodeficiency, and chronic inflammation. Human T-cell leukemia virus type 1 (HTLV-1), hepatitis C virus (HCV), Human herpesvirus 8, and *Helicobacter pylori* have been linked to numerous NHL subtypes [13-15]. Non-Hodgkin lymphoma (NHL) has also been linked to certain drugs, chemical substances, pesticides, wood preservatives, solvents, radiation, and chemotherapy. Congenital immunodeficiency diseases including Wiskott-Aldrich syndrome and severe combined immunodeficiency disease (SCID) and immunosuppressant-induced immunodeficiency states increase risk. AIDS patients may develop primary central nervous system (CNS) lymphoma. Autoimmune diseases such as Sjögren syndrome, rheumatoid arthritis, Hashimoto thyroiditis, and celiac disease increase non-Hodgkin lymphoma risk. The NHL's multifactorial nature is shown by its complex interactions [13-15].

*Clinical Representation*****

This section provides a complete analysis of the earliest indications and symptoms commonly observed in cases of childhood NHL. The common signs and symptoms are ongoing weight loss, headaches, vomiting, heightened swelling and pain in the skeletal system, the existence of a lump or mass in the abdominal or cervical region, the occurrence of excessive bleeding, frequent infections, a whitish hue in the eye, nausea, persistent pallor, alterations in eye or vision function, recurring or enduring fever accompanied by joint swelling, bleeding gums, and nose, as well as swollen ocular globes [16]. Recognition and comprehension of these clinical presentations have significant relevance for the early identification and prompt intervention of pediatric cancer cases, with the ultimate goal of enhancing patient outcomes. 

*Diagnostic Approach*****

Non-Hodgkin Lymphoma (NHL) diagnosis involves clinical assessment, imaging, and laboratory tests. Globally, diagnostic guidelines ensure accurate staging and effective treatment planning. Histopathology, immunohistochemistry, ultrasonography, radiography, and CT are utilized to diagnose lymphoma. Unexplained anemia, thrombocytopenia, leukopenia, hyperuricemia, and high serum lactate dehydrogenase (LDH) [17] are key laboratory markers for identifying children's malignancy, followed by child NHL. The preceding technologies can identify lymphoma and create patient-specific treatment strategies by assessing tumor mass cellular makeup. Malignant Hodgkin and Reed/Sternberg (HRS) cells or large mononuclear cell variations are hallmarks of HL diagnosis. These unique cellular properties illuminate illness progression and nature.

Medical professionals can understand lymphomas and make informed patient care decisions using histology. This thorough approach improves patient outcomes by ensuring accurate diagnosis and successful therapies. Lymphoma children were investigated through histopathology and immunohistochemically in 2002. About 71.40% of children had mixed-cellularity Hodgkin lymphoma (HL), 14.30% had indeterminate, and seven had nodular lymphocyte-predominant Hodgkin lymphoma (NLPHL). About 60% and 40% of 10 children had Burkitt and lymphoblastic lymphoma, respectively. NHL-B and NHL-T cells were 50% immunohistochemically [18]. Abdominal discomfort is often assessed using ultrasound, radiography, and CT scans. These diagnostic tests can detect neck, chest, and abdominal tumors or lymphadenopathy. Childhood NHL differs from HL in that it can affect distant locations [19].

To accurately diagnose NHL in children, pediatric oncology requires a thorough staging approach. Normal staging guidelines recommend contrast-enhanced CT imaging of the neck, chest, abdomen, and pelvis. This procedure helps analyze the disease's breadth and determine the best treatment. Healthcare practitioners can improve childhood NHL diagnosis and staging by using Positron emission tomography (PET) or CT scans. This novel method allows clinicians to customize treatment by assessing the condition more thoroughly. Integrating PET/CT scanning improves diagnostics and patient outcomes [20]. The pediatric department performed contrast-enhanced computerized tomography of the neck, chest, abdomen, and pelvis with or without integrated PET, bilateral iliac crest bone marrow aspiration and biopsy, and lumbar puncture for cerebrospinal fluid examination before starting treatment [21].

Staging and Classification of NHL in Pediatric and Adolescent Groups

Pediatric NHL is classified into four stages using the Murphy Staging from St. Jude Children's Research Hospital [22]. First, restricted-stage lymphomas include stages I and II. Stage I lymphoma affects one tumor and no lymph nodes. No abdominal or chest lymphoma [22]. One tumor and one region of lymph nodes affect stage II lymphomas. The lymphoma may have originated in the digestive tract and spread to lymph nodes above or below the diaphragm [22]. The thymus, central chest lymph nodes, or lung lining typically initiate stage III lymphoma. The abdomen may have started them and spread them. Stage III lymphomas may show tumors above or below the diaphragm, in bones, or on the skin. Multiple lymph nodes above and below the diaphragm and a bone tumor around them are probable [19]. Stage IV lymphoma affects the brain, spinal cord, bone marrow, and CNS. Age, blood LDH level, lymphoma site, and beginning treatment affect childhood NHL outcomes. Better outcomes for younger children. This staging approach predicts and plans pediatric NHL treatment [22]. Table 1 displays WHO's histopathological classifications of pediatric NHL [22] with immunophenotype and common clinical and molecular findings.

**Table 1:** Histopathological categories pediatric NHL by WHO. The table categorizes various non-Hodgkin's lymphoma (NHL) types [22]. WHO: World Health Organization, MYC: MYC proto-oncogene, basic helix–loop–helix (bHLH) transcription factor, TCF3: transcription factor 3, ID3: inhibitor of DNA binding 3, HLH protein: helix–loop–helix proteins, CCND3: cyclin D3, TP53: tumor protein P53, IRF4: interferon regulatory factor 4, CIITA: class II major histocompatibility complex transactivator, TNFAIP3: tumor necrosis factor alpha-induced protein 3, SOCS1: suppressor of cytokine signaling 1, PTPN11: protein tyrosine phosphatase non-receptor type 11, STAT6: signal transducer and activator of transcription 6, ALK: anaplastic lymphoma kinase, NPM: nucleophosmin (also known as NPM1: nucleophosmin 1), TNFRSF14: tumor necrosis factor receptor superfamily member 14, MAP2K1: mitogen-activated protein kinase kinase 1.

WHO classification	Immunophenotype	Clinical presentation	Chromosome abnormalities	Genes affected
Burkitt lymphoma	Mature B-cell	Intra-abdominal (sporadic), head and neck (non-jaw, sporadic), jaw (endemic), bone marrow, CNS	t(8;14)(q24;q32), t(2;8)(p11;q24), t(8;22)(q24;q11)	MYC, TCF3, ID3, CCND3, TP53
Burkitt-like lymphoma with 11q aberration (provisional)	Mature B-cell	Nodal	11q alteration, no MYC rearrangement	
Large B-cell lymphoma with IRF4 rearrangement	Mature B-cell	Nodal (typically head and neck)	Cryptic IRF1 rearrangement with immunoglobulin heavy (IGH) locus	IRF4
Diffuse large B-cell lymphoma	Mature B-cell	Nodal, abdominal, bone, primary CNS (when associated with immunodeficiency), mediastinal	No consistent cytogenetic abnormality identified	
Primary mediastinal (thymic) large B-cell lymphoma	Mature B-cell, often CD30+	Mediastinal, but may also have other nodal or extranodal disease (i.e., abdominal, often kidney)	9p and 2p gains	CIITA, TNFAIP3, SOCS1, PTPN11, STAT6
ALK-positive large B-cell lymphoma		Generalized lymphadenopathy, bone marrow in 25%	t(2;5)(p23;q35); less common variant translocations involving ALK	ALK, NPM
T-lymphoblastic leukemia/lymphoma	T lymphoblasts (TdT, CD2, CD3, CD7, CD4, CD8)	Mediastinal mass, bone marrow		
B-lymphoblastic leukemia/lymphoma	B lymphoblasts (CD19, CD79a, CD22, CD10, TdT)	Skin, soft tissue, bone, lymph nodes, bone marrow		
Pediatric-type follicular lymphoma	Mature B-cell	Nodal (typically head and neck)		TNFRSF14, MAP2K1
Pediatric nodal marginal zone lymphoma	Mature B-cell	Nodal (typically head and neck)		

*Treatment of Pediatric Non-Hodgkin Lymphoma*****

Immunotherapy, targeted therapies, small-molecule inhibitors, radioimmunotherapy, stem cell transplantation, precision medicine, and combination drugs are emerging NHL treatments. The NHL histologic subtype determines juvenile lymphoma treatment by a multidisciplinary team of cancer specialists. Pediatric oncologists and hematologists plan NHL treatment for children. They start with chemotherapy and other treatments. Rehabilitation experts assist children in restoring strength and mobility by working with the medical team to address physical concerns during treatment, after treatment, or during NHL maintenance. Pediatric nurses are also important for NHL management. NHL children receive specialized and supportive care. Nurses, social workers, and non-governmental organizations can aid pediatric NHL patients by providing comfort and emotional support during their journey [23]. Two recently constructed primary public hospitals specialize in pediatric oncology; however, there are few nationwide. The healthcare system, including cancer detection, treatment, and management, has severe problems due to a lack of infrastructure and educated healthcare workers.

The lack of public pediatric oncology centers shows Bangladesh's need for better healthcare. This shortage limits access to specialized care for children with cancer and strains hospitals, causing overcrowding and resource strain. The lack of infrastructure and educated health workers further complicates the healthcare system of Bangladesh. Lack of advanced diagnostic technology and specialist treatment centers delays cancer diagnosis. Oncologists, nurses, and support staff are scarce, making it difficult to provide comprehensive and effective care to pediatric cancer patients. Bangladesh has 20-22 cancer service centers for 170 million people, which is low [4] and 500 hospital beds for cancer patients (adults and children), but few pediatric hematologists and oncologists [2]. Parents and families continue to suffer the terrible effects of childhood cancer, as seen by greater impact scores in all negative domains. Single motherhood, lower income, and longer medical travel distances increase this impact [24].

## Review

Bangladesh has a higher incidence of childhood cancer, with 13,000 new cases reported annually [25]. In Bangladesh, from 2001 to 2014, NHL (59%) was more common than HL (41%) in a study on 3,143 children [26]. The families of children affected by childhood cancer endure elevated levels of anxiety, depression, psychological distress, financial strain, and constant worry for their children's well-being [27]. Male children were the majority, accounting for 60.7% of the participants (out of 272) [28]. Additionally, a significant proportion of the children came from rural areas (67.5%) and belonged to nuclear families (60.7%). It is worth noting that approximately 27.7% of the fathers worked as farmers, while most mothers (94.2%) were engaged in household duties as housewives [28]. However, the true extent of childhood cancer's impact on families in the country remains unknown because of the unavailability and insufficiency of studies, surveys, and journals. 

Methodology

**Figure 1:**
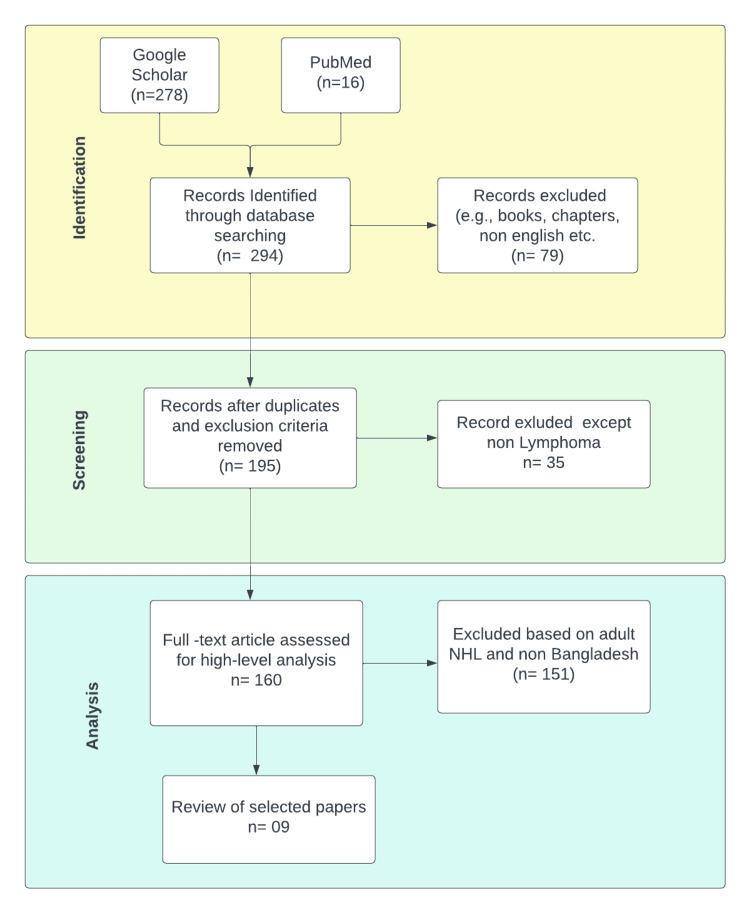
Preferred Reporting Items for Systematic Reviews and Meta-Analyses (PRISMA) flow chart of research conducted on childhood non-Hodgkin lymphoma (NHL) in Bangladesh, 2018-2023.

This review paper used the Preferred Reporting Items for Systematic Review and Meta-Analysis (PRISMA) 2020 standards as a framework for conducting the systematic review. We conducted a comprehensive search on two electronic databases, namely Google Scholar and PubMed, covering the period from 2018 to 2023. The search terms included in this study encompassed the geographical location of Bangladesh and the medical conditions of pediatric NHL. This study encompasses research undertaken within Bangladesh, focusing on individuals under 19 and containing relevant information about NHL. We have omitted papers that are not authored in the English language. On August 8, 2023, a search was conducted on Google Scholar and PubMed. In the initial phase, a total of 278 papers were obtained from Google Scholar, while 16 papers were retrieved from PubMed. The elimination of duplicate papers, and non-English was conducted on both ends. The exclusion of articles undertaken in other countries was determined by reviewing the titles and abstracts. Additionally, we have identified NHL case studies that were conducted on adult individuals and have chosen to omit them from our analysis. Our research mostly centered on pediatric non-Hodgkin lymphoma (NHL) in the context of Bangladesh. In total, nine publications were obtained from the aforementioned investigations, comprising two case studies and the remaining papers focusing on categorizing pediatric NHL patients. Figure 1 shows the step-by-step procedure that has been followed using PRISMA. The flow chart shows the systematic review's paper screening and selection process. The general prevalence of cancer was higher in males than females in Bangladesh [2]. Our review also showed that, according to the selected paper, the number of pediatric NHLs is higher than the HL.

Result

In this section, we are going to discuss the summary of the frequency of NHL, HL, and NHL ratios, along with the male-female children ratio with NHL. Also, the cost of the NHL treatment is going to be discussed later in the section, followed by a detailed analysis of the selected seven articles and two case studies.

**Figure 2:**
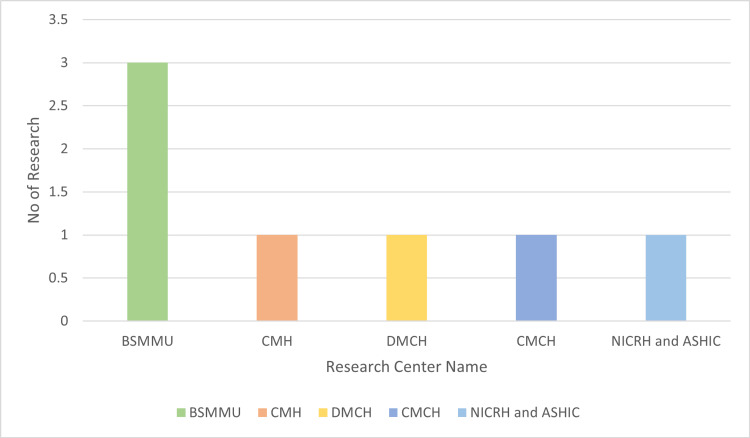
Research conducted in Bangladesh (2018–2023). The provided column chart shows a visual representation of the quantity of research studies focused on pediatric non-Hodgkin lymphoma (NHL) completed by five distinct institutes during the period spanning from 2018 to 2023 [2,3,29-33]. BSMMU: Bangabandhu Sheikh Mujib Medical University, CMH: Combined Military Hospital (CMH), DMCH: Dhaka Medical College Hospital, CMCH: Chittagong Medical College Hospital, and NICRH: National Institute of Cancer Research and Hospital with ASHIC Foundation.

**Figure 3:**
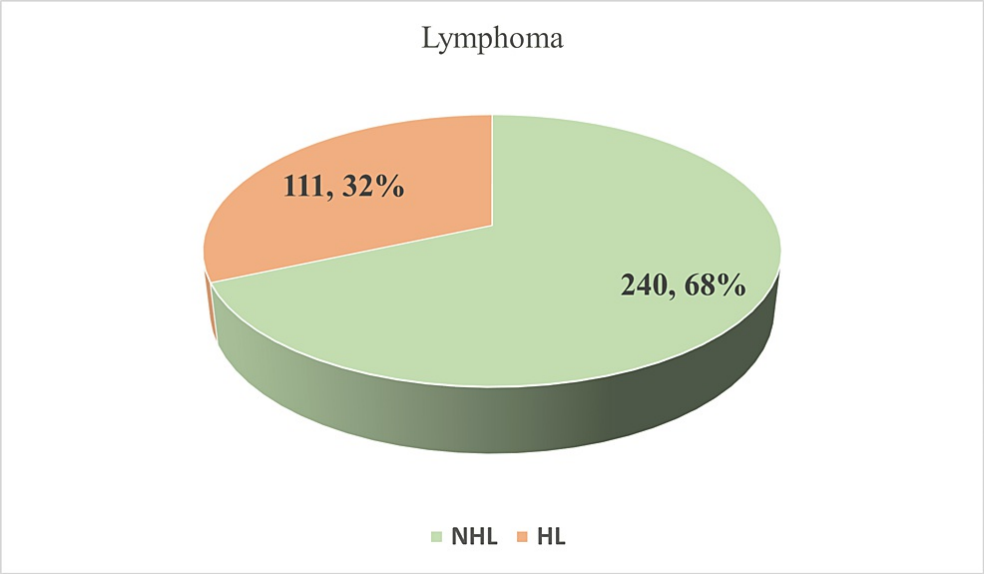
Pediatric NHL and HL frequency (N = 351) The given pie chart provides a visual representation of the distribution of lymphoma cases, specifically differentiating between non-Hodgkin lymphoma (NHL) and Hodgkin lymphoma (HL) categories. In the provided sample, it is seen that NHL cases constitute 32% of the overall total, while HL cases comprise the remaining 68% [2,3,30].

**Figure 4:**
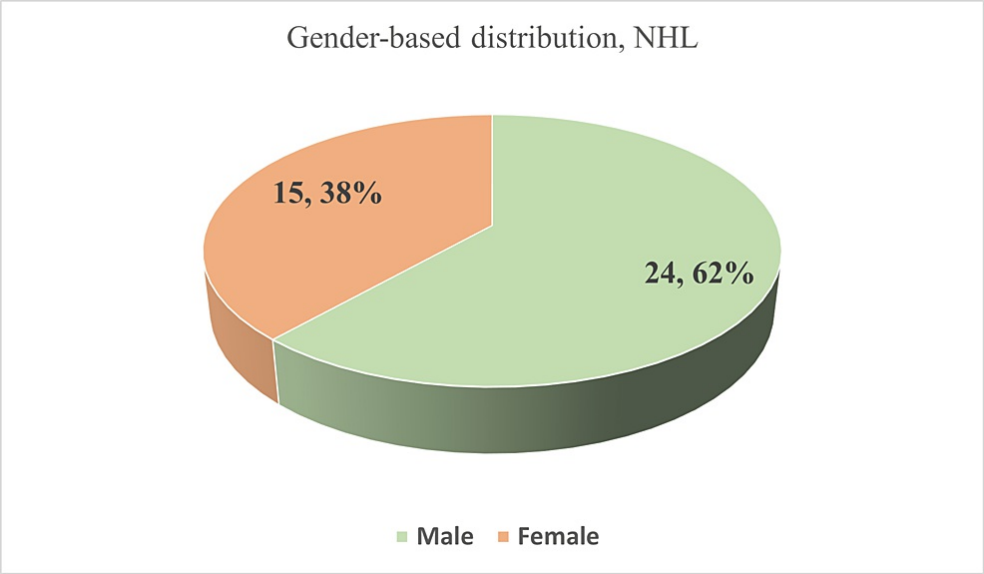
Gender-based distribution of pediatric NHL patients (N = 39). The presented pie chart shows the distribution of male and female children patients with non-Hodgkin lymphoma (NHL) in Bangladesh. Based on the data presented, it is evident that boys comprise 68% of pediatric NHL cases, with girls making up the remaining 32% [3,30,33].

In this paper, we gathered information spanning from 2001 to 2022, based on seven research papers published between 2018 and 2023. Six of the studies have been done in the capital of Bangladesh, Dhaka. Three research studies have been done at Bangabandhu Sheikh Mujib Medical University (BSMMU), Dhaka. The rest of the research centers are Combined Military Hospital (CMH), Dhaka Medical College Hospital (DMCH), Chittagong Medical College Hospital (CMCH), and the National Institute of Cancer Research and Hospital (NICRH) with the ASHIC Foundation. Figure 2 shows the research centers where the research has been conducted. Table 2 shows the frequency of the occurrence of NHL in children. Figure 3 shows that NHL (68%) in children is higher than HL (32%). Also, Figure 4 shows that among the three researchers, the ratio of males (62%) is 1.5 times higher than females (38%).

**Table 2:** Frequency of occurrence of non-Hodgkin lymphoma (NHL) in children, based on the seven research papers. The presented table provides information regarding the timeline, duration, number of patients (N), and frequency of observed outcomes or events for each specified time period of research conducted from 2018 to 2023 from seven research papers [2,3,29-33].

Timeline	Duration	Age	Number of patients (N)	Frequency
2001–2014 [2]	13 Years	Under 20 years	3143	7.92% (249)
2016–2021 [3]	5 Years	Up to 12 years	170	5.88% (10)
2012–2013 [29]	12 Months	Under 12 years	50	16% (8)
2017–2018 [30]	12 Months	0–14 Years	43	23.26% (10)
April 2012–March 2013 [31]	12 Months	1–15 Years	51	29.4% (17)
January 2014–August 2015 [32]	18 Months	7 Months–16.5 years	200	8% (16)
July 2022–December 2022 [33]	6 Months	2.5–15 Years	90	11.11% (19)

Epidemiology of NHL in pediatric patients in Bangladesh

In the case of Bangladesh, the number of large-scale epidemiological studies regarding the NHL and even childhood cancer is insufficient [6].

In a study conducted by Choudhury et al. (2019), data was gathered on malignant neoplasms in individuals under the age of 20 who were diagnosed between 2001 and 2014 [2]. The data, consisting of 3143 cases, was obtained from the NICRH and ASHIC Foundation in Bangladesh. The analysis focused on examining the age pattern and distribution of various cancer forms and calculating the corresponding incidence rates afterward. The findings indicate that within the age group of 0-14, a total of 220 individuals were affected by non-Hodgkin lymphoma (NHL), a figure that is nearly twice as high as the number of individuals affected by Hodgkin lymphoma (HL), which stood at 103. A comparable trend is observable between the ages of 15 and 20, when the NHL (29) exhibits double the number of patients as the HL (15) at this specific time frame.

A study conducted by Nahar et al. (2021) to investigate the distribution of lymphoma in a sample of 170 children from 2016 to 2021 in the Combined Military Hospital (CMH) [3]. Of the total patient population, 102 individuals (60%) were diagnosed with hemopoietic cancer, while 68 individuals (40%) were diagnosed with solid tumors. Leukemia was found to be the most prevalent type of cancer in children, accounting for 52.4% of cases. Additionally, central nervous system (CNS) tumors were observed in 12% of cases, while lymphoma accounted for 10% of cases. Childhood lymphoma is a combination of hematopoietic cancer, accounting for 60% of cases, and solid tumors, accounting for the other 40% of cases. Out of a total of 170 pediatric cancer patients, 17 individuals were found to have been diagnosed with lymphoma. Among these cases, ten patients (4F, 58.8%) were identified as having NHL, while the remaining seven patients (4F, 41.2%) were diagnosed with HL.

Rahman et al. (2020) reported a study in 2012-2013 on 50 patients aged not more than 12 years at pediatric hematology and oncology, Dhaka Medical College Hospital (DMCH), Dhaka [29]. They focused on age and gender distribution, symptoms, the final diagnosis, and complications of bone marrow aspiration. This prospective observational study aimed to assess the morbidities associated with bone marrow aspiration in pediatric patients and examine the potential relationship between these morbidities and the various sites of aspiration. The final diagnosis showed that 16% (eight out of 50) suffered from NHL.

Rahman et al. (2020) conducted another study in 2017-2018 where a total of 43 cases of children aged (0-14 years) with solid malignant tumors were analyzed over one year at Chattogram Medical College Hospital (CMCH), Chattogram [30]. The prevailing tumor type observed in this study was lymphoma, specifically NHL, which exhibited a greater prevalence than other tumor types. A total of 25.3% of the observed tumors were identified as lymphomas. Out of the total number of instances, ten were diagnosed as NHL, whereas only one was identified as Hodgkin lymphoma. The average age is 8. In the age bracket of 0-4 years, the male population exceeds the female population by a ratio of 9 to 1.

Begum et al. (2019) conducted a study in 2019 that was carried out at the Department of Pediatric Hematology and Oncology, Bangabandhu Sheikh Mujib Medical University (BSMMU), Bangladesh, during the period from April 2012 to March 2013 [31]. The research encompassed a cohort of 51 pediatric patients, ranging in age from 1 to 15 years, including both genders, who had been clinically diagnosed with several forms of hematological malignancies. Diarrhea was observed in these pediatric patients at some stage during their stay. Diarrhea is characterized by the occurrence of a minimum of three loose or watery bowel movements during a 24-hour period. Out of the patients examined, 29.4% were identified as having NHL.

The objective of the study conducted by Doherty et al. (2020) was to examine the symptoms, treatments, and outcomes experienced by pediatric cancer patients who underwent pediatric palliative care under the supervision of a professional team at a publicly funded tertiary hospital in Bangladesh [32]. A retrospective analysis was performed utilizing data extracted from the Pond4Kids online database, encompassing all pediatric patients who were under the care of the Pediatric Palliative Care (PPC) team within the Department of Pediatric Hematology and Oncology at BSMMU during the period spanning from January 2014 to August 2015, ranging in age from seven months to 16.5 years. The study sample consisted of children diagnosed with different forms of cancer, with NHL being the most prevalent (n = 16 out of 200; 8.0%). The team of experts in the field of pediatric palliative care delivered specialized treatments to these children, with a primary emphasis on effectively managing symptoms and enhancing their overall quality of life.

The goal of the study conducted by Akter et al. (2023) was to identify and analyze a group of pediatric oncology patients who were admitted with febrile neutropenia throughout the period from July 2022 to December 2022 at BSMMU [33]. The average age of the patients was 6.9 years, with an age range of 2.5 years to 15 years, with 57 identified as male and 33 identified as female. In the male patient cohort, 15.78% of individuals received a diagnosis of NHL; however, in the female patient cohort, the prevalence of NHL cases was comparatively higher at 30.30%.

Besides the above-mentioned articles, we found two case studies (in 2019 and 2021) on childhood NHL in Bangladesh. Hossain et al. (2021) reported a case study on a 14-year-old boy with primary CNS lymphoma (PCNSL), a type of NHL that arises in the central nervous system [34]. According to the study, this is the first instance of primary central nervous system non-Hodgkin lymphoma (PCNSL) that affected the lateral, third, and periventricular regions. They also mentioned that the AIDS pandemic increased the prevalence of PCNSL in immunocompromised and older people. Hyper-density on CT scans and hypo-intensity to iso-intensity on T1-weighted MRI imaging demonstrate uniform contrast enhancement in non-AIDS patients. Central neurocytoma, meningioma, ependymoma, choroid plexus papilloma, and metastases are intraventricular CNS lesion differentials. PCNSL is treated with systemic or intrathecal chemotherapy and radiotherapy, not surgery. A restricted biopsy can diagnose problems, reducing side effects [34]. In the case study reported by Hafiz et al. (2019), a two-year-old male child was brought to the Department of Pediatric Hematology and Oncology at BSMMU. He had been diagnosed with NHL, which arose from the abnormal growth of lymphoid cells [35].

Healthcare management, infrastructure, and expenses for NHL in Bangladesh

In Bangladesh, the first pediatric oncology center at BSMMU was created in the early 1990s. The pediatric oncosurgery department was established in Dhaka Medical College Hospital (DMCH) and Chattogram Medical College Hospital (CMCH) in 2017.

**Figure 5:**
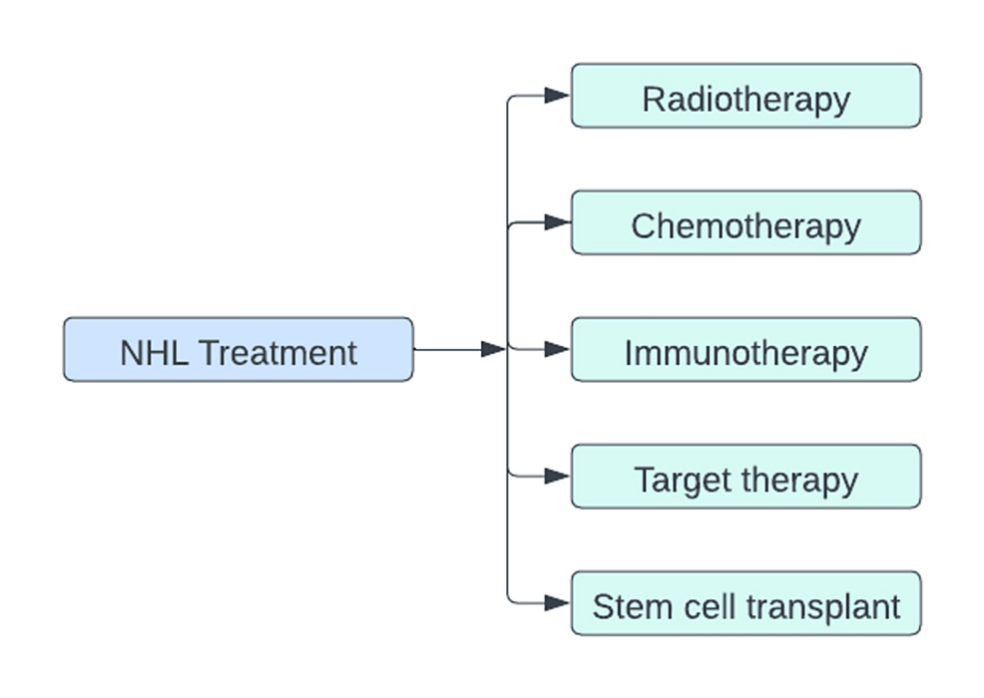
NHL treatment available in Bangladesh. Note: This image is the author's own creation.

In 2005, only solid tumor treatment was provided at the National Institute of Cancer Research and Hospital (NICRH). DMCH opened its doors as the first government-run pediatric hematology and oncology center in 2010. The adoption of protocol-based care at Dhaka Shishu Hospital and the tireless efforts of Chattogram Medical College Hospital, Sir Salimullah Medical College Hospital, and Sylhet MAG Osmani Medical College Hospital have revolutionized care since 2014. Pediatric cancer patients are actively treated at private cancer treatment facilities like Ahsania Mission Cancer Hospital, Square Hospital, Delta Medical College Hospital, and United Hospital; however, the scarcity of pediatric oncologists poses a challenge. These hospitals provide the best possible care for young cancer patients by prioritizing the recruitment and collaboration of pediatric oncologists, ultimately raising the likelihood of their recovery and general well-being [25]. The National Institute of Laboratory Medicine and Referral Center (NILMRC) started its journey in 2019 to provide pathologic services and utilize the institute as a referral laboratory. Figure 5 shows the treatments that are available in Bangladesh currently [33].

Table 3 describes the protocol followed for pediatric NHL in Bangladesh [22]. This protocol includes several treatment groups, such as mature B-cell NHL, lymphoblastic lymphoma, anaplastic large-cell lymphoma, lymphoproliferative disease associated with immunodeficiency, and rare NHL have been reported here.

**Table 3:** Treatment options for childhood non-Hodgkin lymphoma (NHL). SCT: Stem cell transplant, CAR T-cell therapy: chimeric antigen receptor T-cell therapy, CNS: central nervous system, PTLD: post-transplant lymphoproliferative disorder, MALT lymphoma: mucosa-associated lymphoid tissue lymphoma, EBV: Epstein-Barr virus. Source: [22].

Treatment Group		Treatment Options	
		Newly diagnosed	Recurrent or refractory
Mature B-cell NHL	Burkitt lymphoma/leukemia	Surgery (for stage I and II only), Chemotherapy with or without rituximab	Chemotherapy with or without rituximab, Allogeneic or autologous SCT Bispecific antibody CAR T-cell therapy
	Diffuse large B-cell lymphoma	Surgery (for stage I and II only), Chemotherapy with or without rituximab	Chemotherapy with or without rituximab, Allogeneic or autologous SCT, Bispecific antibody, CAR T-cell therapy
	Primary mediastinal B-cell lymphoma	Chemotherapy and rituximab	
Lymphoblastic lymphoma		Chemotherapy, Cranial radiation, and therapy for overt CNS disease only	Nelarabine or nelarabine-containing chemotherapy regimens, Chemotherapy, Bortezomib with chemotherapy, Allogeneic SCT
Anaplastic large cell lymphoma		Surgery followed by chemotherapy (for stage I), Chemotherapy	Chemotherapy, brentuximab, and/or ALK inhibitors (e.g., crizotinib or alectinib), Allogeneic or autologous SCT
Lymphoproliferative disease associated with immunodeficiency	Lymphoproliferative disease associated with primary immunodeficiency	Chemotherapy with or without rituximab, Allogeneic SCT	
	NHL associated with DNA repair defect syndromes	Chemotherapy	
	HIV-associated NHL	Chemotherapy with or without rituximab	
	PTLD	Surgery and reduction of immunosuppressive therapy, if possible Rituximab alone, Standard or slightly modified chemotherapy with or without rituximab (for B-cell PTLD), Low-dose chemotherapy with or without rituximab (for EBV-positive B-cell PTLD)	
Rare NHL	Pediatric-type follicular lymphoma	Surgery only, Chemotherapy with or without rituximab	
	Marginal zone lymphoma	Surgery only, Radiation therapy, Rituximab with or without chemotherapy, Antibiotic therapy, for MALT lymphoma	
	Primary CNS lymphoma	Chemotherapy, Radiation therapy	
	Peripheral T-cell lymphoma	Chemotherapy, Radiation therapy, Allogeneic or autologous SCT	
	Cutaneous T-cell lymphoma	No standard treatments have been established	
	Mycosis fungoides	No standard treatments have been established	

NHL diagnosis: Investigation costs in Bangladesh

To address the challenges of treatment costs, the national health policy of Bangladesh has proposed a significant increase in budget allocation for healthcare, aiming to achieve healthcare facilities for all and ensure health equity and financial risk protection [22]. The exorbitant cost of treating childhood cancer places an immense financial burden on families. Table 4 shows some primary investigation expenses needed to diagnose NHL as reported by the National Institute of Laboratory Medicine and Referral Center (NILMRC), and the total cost is approximately USD 348. The full list is available on the NILMRC website [36]. 

**Table 4:** Price of tests required for NHL treatment proposed by the National Institute of Laboratory Medicine and Referral Center. S. iron: serum iron, S. LDH: serum lactate dehydrogenase, CBC: complete blood count, CBC with PBF: complete blood count with peripheral blood film, FNAC: fine needle aspiration cytology, CT scan: computed tomography scan, USG: ultrasonography, MRI: magnetic resonance imaging, MRA: magnetic resonance angiography, MRS: magnetic resonance spectroscopy, MRV: magnetic resonance venography, TSH: thyroid-stimulating hormone. Source: [33].

S. no	Investigations	Price (USD)
1.	S. Iron	2.28
2.	S. LDH	3.19
3.	Thyroid profile (T3, T4, TSH)	2.73
4.	Bone marrow study	9.1
5.	CBC	1.37
6.	CBC with PBF	2.73
7.	CD-15, 20, 3, 30, 34, 45, 5, 99	22.75 (each)
8.	FNAC	1.37
9.	Histopathology (small or large)	1.37–2.73
10.	Histopathology slide review	2.73
11.	CT scan	18.2
12.	USG	1.00–2.00
13.	MRI of the brain (without contrast), MRI of the brain (with contrast), MRI of the brain with MRA (without contrast), MRI of the brain with MRS (without contrast), MRI of brain MRV (without contrast), MRI of cervical spine (without contrast) MRI of dorsal spine (without contrast), MRI of lumbar spine (without contrast)	27.30–36.40
14.	MRI of the whole spine (without contrast)	81.9

There are about 250,000 cancer patients nationwide, according to the National Institute of Cancer Research and Hospital (NICRH), yet only 50,000 of them routinely receive treatment [37]. A single cancer patient requires roughly USD 5,824.65 per year [37], whereas the gross domestic product (GDP) of Bangladesh is USD 2,940.58 in 2022, as reported by the World Bank [38]. This substantial financial burden stems from several factors in Bangladesh. Firstly, advanced medical technologies and the specialized equipment required to diagnose and treat pediatric cancer necessitate significant investment. Additionally, the complex nature of cancer treatment for children demands a multidisciplinary approach involving numerous healthcare professionals, such as oncologists, surgeons, radiologists, and nurses. The cost further escalates due to the need for extensive long-term follow-up care and rehabilitation programs tailored specifically for young patients. Furthermore, research and development in pediatric oncology are vital to continuously improving treatment outcomes and developing novel therapies; however, these endeavors add to the overall expenses [22,33,36-38].

The management of pediatric cancer in Bangladesh is encountering significant obstacles and challenges due to a notable increase in patient referrals to Pediatric Oncology Centers in recent times and a significant prevalence of reluctance among individuals to undergo treatment. Although Bangladesh has taken several necessary steps to combat childhood cancer, studies show that 54.3% of medical tourists traveled from Bangladesh to India in 2020 [39], where a large portion of childhood cancer is diagnosed [39]. 

Discussion

We employed PRISMA in this paper to provide an overview of the state of pediatric NHL in Bangladesh based on research conducted from 2018 to 2023. Our search terms led us to seven research papers and two study cases. However, the research papers we examined do not solely focus on pediatric NHL. The two study cases are based on two pediatric NHL patients. The results demonstrate that there are more pediatric NHL patients than pediatric HL patients. Additionally, there are more male patients than female ones. The general infrastructure and cost of treating NHL in Bangladesh are also discussed in this study. The prevalence of HL in children is higher globally than it is in Bangladesh. In Bangladesh, the data on childhood cancer is not officially reported because there is a lack of countrywide cancer registries and surveillance systems. Also, the national guidelines have not been updated since 2017. The hard copy of the national cancer guideline for Bangladesh is available; however, it is not available on authentic online platforms or reputed journals. The investigation of childhood cancer, specifically NHL, in Bangladesh is hindered by the presence of unregistered data and insufficient information gathering. The inadequate portrayal of current data compounds the difficulties encountered by researchers and healthcare practitioners. The absence of regular and systematic follow-up, along with the limited availability of reliable information on credible websites, exacerbates the challenges associated with obtaining pertinent and up-to-date data. Furthermore, it is evident that the current research and data collection pertaining to childhood cancer are insufficiently current, indicating a systemic delay throughout the discipline. The aforementioned issues are notably conspicuous when examining childhood NHL, given the scarcity of dedicated research pertaining to this topic in Bangladesh. The confluence of these variables poses obstacles to conducting a thorough examination of childhood NHL within the nation, thus constraining the capacity to devise focused therapies, comprehend epidemiological patterns, and enhance overall patient care and results.

Therefore, sufficient research is required to combat this disease. Bangladesh's government is trying to improve the infrastructure and provide better facilities to cancer patients, including NHL, but there is a lack of information sharing between clinicians and researchers. It is also observed that there is a lack of awareness among general people about NHL due to the small extent of knowledge among health workers. This results in NHL patients, especially their guardians, getting confused or unknowingly ignoring this serious condition. Identifying and properly treating childhood cancer necessitates a solid foundation of accurate diagnosis and extensive research. It is essential to bridge the gap between available data and data-driven research, particularly in areas such as NHL in children. By dedicating resources and efforts to comprehensive studies, we can enhance our understanding and develop more effective treatment approaches, ultimately offering hope and improved outcomes for young cancer patients. Every year, many individuals travel to India seeking treatment for childhood cancer. The provision of comprehensive and effective care to pediatric cancer patients is hindered by the shortage of adequately informed healthcare professionals about NHL, including physicians, nurses, and support staff. Efforts should be made to attract and retain highly trained healthcare professionals to be involved in research work, providing them with the necessary resources and incentives to specialize in pediatric oncology.

## Conclusions

This review paper provides an overview of the recent scenario for pediatric NHL patients in Bangladesh based on the published papers from 2018 to 2023. It is imperative to conduct thorough and comprehensive research to enhance comprehension of the attributes of NHL and develop effective strategies for combating this ailment. Furthermore, during data representation, it is essential to gather all available data on age, gender, family history, symptoms, and lifestyle. For instance, of the seven studies, only three referenced the ratio of male and female children. By prioritizing the development of pediatric oncology services and bolstering independent, identical case studies, we can significantly improve the quality of care for children battling cancer and build up a strong database of pediatric NHL patients in Bangladesh for further research. The primary constraint of this study pertains to the scarcity of online research resources accessible for reference. Most of the data presented in the chosen studies originates from the timeframe spanning 2000 to 2017, a range that is deemed insufficient and antiquated. As a result, precisely interpreting the present state of pediatric non-Hodgkin's lymphoma (NHL) becomes challenging. In our next research, our objective is to do a thorough examination of the cost-effectiveness of NHL treatment, with a particular focus on analyzing the complex financial aspects related to insurance coverage, government funding, donations, and other financing mechanisms that have an influence on patient care and outcomes.
